# The Prebiotic Inulin Aggravates Accelerated Atherosclerosis in Hypercholesterolemic APOE*3-Leiden Mice

**DOI:** 10.3390/nu10020172

**Published:** 2018-02-03

**Authors:** Lisa R. Hoving, Margreet R. de Vries, Rob C. M. de Jong, Saeed Katiraei, Amanda Pronk, Paul H. A. Quax, Vanessa van Harmelen, Ko Willems van Dijk

**Affiliations:** 1 Department of Human Genetics and Einthoven Laboratory for Experimental Medicine, Leiden University Medical Center (LUMC), 2300 RC Leiden, The Netherlands; S.Katiraei@lumc.nl (S.K.); A.C.M.Pronk@lumc.nl (A.P.); V.J.A.van_Harmelen@lumc.nl (V.v.H.); 2 Department of Surgery and Einthoven Laboratory for Experimental Medicine, Leiden University Medical Center (LUMC), 2300 RC Leiden, The Netherlands; M.R.de_Vries@lumc.nl (M.R.d.V.); R.C.M.de_Jong@lumc.nl (R.C.M.d.J.); P.H.A.Quax@lumc.nl (P.H.A.Q.); 3 Department of Medicine, Division Endocrinology, Leiden University Medical Center (LUMC), 2300 RC Leiden, The Netherlands

**Keywords:** atherosclerosis, inulin, prebiotics, APOE*3-Leiden mice, cholesterol, remodeling

## Abstract

The prebiotic inulin has proven effective at lowering inflammation and plasma lipid levels. As atherosclerosis is provoked by both inflammation and hyperlipidemia, we aimed to determine the effect of inulin supplementation on atherosclerosis development in hypercholesterolemic APOE*3-Leiden (*E3L*) mice. Male *E3L* mice were fed a high-cholesterol (1%) diet, supplemented with or without 10% inulin for 5 weeks. At week 3, a non-constrictive cuff was placed around the right femoral artery to induce accelerated atherosclerosis. At week 5, vascular pathology was determined by lesion thickness, vascular remodeling, and lesion composition. Throughout the study, plasma lipids were measured and in week 5, blood monocyte subtypes were determined using flow cytometry analysis. In contrast to our hypothesis, inulin exacerbated atherosclerosis development, characterized by increased lesion formation and outward vascular remodeling. The lesions showed increased number of macrophages, smooth muscle cells, and collagen content. No effects on blood monocyte composition were found. Inulin significantly increased plasma total cholesterol levels and total cholesterol exposure. In conclusion, inulin aggravated accelerated atherosclerosis development in hypercholesterolemic *E3L* mice, accompanied by adverse lesion composition and outward remodeling. This process was not accompanied by differences in blood monocyte composition, suggesting that the aggravated atherosclerosis development was driven by increased plasma cholesterol.

## 1. Introduction

Atherosclerosis is a chronic inflammatory disease of the arteries, which may ultimately prevent adequate blood flow to target tissues leading to cardiovascular complications including heart attack and stroke. In modern society, atherosclerosis is a leading cause of death [1]. An important risk factor for atherosclerosis is increased plasma low-density lipoprotein (LDL) cholesterol. Accumulation and modification of LDL in the arterial wall lead to activation of endothelial cells and increased influx of monocytes. These processes initiate local inflammation characterized by production of pro-inflammatory chemokines and cytokines leading to foam cell formation [2,3]. Foam cell formation leads to proliferation and migration of vascular smooth muscle cells (SMCs) and extracellular matrix deposition. These events ultimately result in intimal hyperplasia and vascular remodeling [4].

Atherosclerosis development can be attenuated by reducing LDL levels and inflammation pharmacologically, for instance, by using statins [5]. However, statin treatment prevents roughly 30% of all cardiovascular events [6], leaving ample opportunities for additional treatment strategies. Epidemiological studies have shown that diets rich in fibers are associated with reductions in the risk of cardiovascular diseases and atherosclerosis development [7,8,9,10]. 

A category of dietary fibers that received great attention in the last decade is inulin-type fructans. Inulin is a dietary fiber that meets the three classification criteria for being considered as a prebiotic [11], i.e., it is resistant to hydrolysis by human enzymes and therefore minimally absorbed in the gastrointestinal tract, it is fermented by colonic microbiota, and it selective stimulates the growth and/or activity of beneficial colonic bacteria. 

Evidence is increasingly indicating that inulin exerts favorable effects on a variety of immune-related diseases, such as inflammatory bowel disease [12,13], rheumatoid arthritis [14], and on low-grade chronic inflammation that is associated with cardiovascular disease in humans [15,16]. Furthermore, there are indications that inulin has beneficial effects on hyperlipidemia in rodents [17,18]. However, data on the effects of inulin on lipid metabolism in humans are inconsistent [19,20,21,22,23,24]. The previous studies in rodents showing beneficial effects of inulin on hyperlipidemia and atherosclerosis were performed in LDL-receptor knockout mice and APOE-deficient mice, which are both models characterized by severely hampered lipoprotein remnant metabolism. 

We extended these findings and investigated whether inulin may delay or prevent development of atherosclerosis in APOE*3-Leiden (*E3L*) transgenic mice [25]. *E3L* mice are a well-established preclinical mouse model to study interventions aimed to improve lipid metabolism and to decrease atherosclerosis development [26,27]. We studied accelerated atherosclerosis development in these mice after placement of a non-constrictive polyethylene cuff around the femoral artery. The ensuing vascular pathology has been shown to be sensitive to both modulation of plasma cholesterol levels and inflammation, and may thus also be affected by inulin. 

## 2. Materials and Methods

### 2.1. Mice and Diet

Male transgenic APOE*3-Leiden (*E3L*) mice (12–17 weeks of age) were fed a high cholesterol diet containing 1% cholesterol and 0.05% cholic acid (Diet W; AB-Diets, Woerden, The Netherlands) ±10% inulin (FrutaFit HD; Sensus, Roosendaal, The Netherlands) for a total period of 5 weeks (*n* = 11–13 mice per group). Before the experiment, mice were randomized based on age, body weight, and plasma cholesterol levels. During the experiment, 2 mice of the control group (*n* = 13 at the start of the experiment) and 1 mouse of the inulin group (*n* = 14 at the start of the experiment) died due to unspecified reasons unrelated to the study. Mice were housed under temperature- and humidity-controlled conditions with a 12:12 h light-dark cycle and free access to food and water. During the diet intervention, body weight and food intake were measured weekly. Mouse experiments were performed in compliance with Dutch government guidelines and the Directive 2010/63/EU of the European Parliament and had received approval from the University Ethical Review Board (Leiden University Medical Center, Leiden, The Netherlands). 

### 2.2. Cuff-Induced Atherosclerotic Lesion Formation

After 3 weeks on diet W ± 10% inulin, mice were subjected to femoral arterial cuff placement to induce accelerated atherosclerosis development as described previously [27,28]. Briefly, before surgery mice were anesthetized with an intraperitoneal injection of 5 mg/kg Midazolam (Roche, Woerden, The Netherlands), 0.5 mg/kg Medetomidine (Orion, Helsinki, Finland), and 0.05 mg/kg Fentanyl (Janssen, Beerse, Belgium). The right femoral artery was exposed from surrounding tissue, and sheathed with a rigid non-constrictive polyethylene cuff (Portex, 0.40 mm inner diameter, 0.80 mm outer diameter, and an approximate length of 2.0 mm). After the surgery, the anaesthesia of the mice was antagonized with Atipamezol (1.7 mg/kg, Orion) and Fluminasenil (0.3 mg/kg, Fresenius Kabi). Buprenorphine (0.1 mg/kg, MSD Animal Health) was given after surgery to relieve pain. Mice were sacrificed after 5 weeks of dietary intervention, which was 2 weeks after perivascular cuff placement when profound intima formation with signs of atherosclerosis had developed. Before sacrifice, mice were anesthetized with intraperitoneal injection containing a mixture of Midazolam (8 mg/kg)/Fentanyl (0.08 mg/kg)/Dexdomiter (0.8 mg/kg)/NaCl (0.9%) and subsequently euthanized. Orbital blood was obtained for plasma isolation, which was stored at −20 °C until further analysis. The thorax was opened and mild pressure-perfusion (100 mmHg) was performed with ice-cold PBS for 10 min by cardiac puncture in the left ventricle. After perfusion, the cuffed femoral artery was harvested, fixed overnight in 4% formaldehyde in PBS, and finally paraffin-embedded. Serial cross sections (5 µm thick) were used throughout the entire length of the cuffed femoral artery for (immuno)histochemical analysis.

### 2.3. (Immuno)histochemical Staining

As used previously [27,28], histological Weigert’s elastin staining was used to visualize elastic laminae, and a Sirius red staining was performed to quantify the collagen content within the atherosclerotic lesion. The composition of the thickened atherosclerotic lesion was visualized and evaluated by immunohistochemical stainings. Primary antibodies for MAC3 (for macrophages; Rat Anti-Mouse (#550292, BD-Pharmigen, San Diego, CA, USA) 1:300 in 1% PBSA) or α-smooth muscle cell actin (α-SMC) (for smooth muscle cells; Mouse Anti-Human (clone 1A4, #M0851, Dako, Agilent, Amstelveen, The Netherlands) 1:1000 in 1% PBSA) were applied on tissue sections and incubated overnight. After washing with PBS, secondary antibodies for MAC3 (Goat Anti-Rat (#BA-9401, Vector, Burlingame, CA, USA) 1:300 in 1% PBSA) or α-SMC (HRP Horse Anti-Mouse (#PI-2000, Vector, Burlingame, CA, USA) 1:300 in 1% PBSA) were applied, both developed with 3,3’-diaminobenzidine (DAB, #K4007, Dako Agilent, Amstelveen, The Netherlands), and counterstained with hematoxylin. 

### 2.4. Atherosclerotic Lesion Analysis

Six sequential sections were used per vessel segment to quantify atherosclerotic lesion formation based on Weigert’s elastin staining. Using image analysis software (Leica Qwin, Wetzlar, Germany) total cross-sectional lumen area, total cross-sectional medial area between the external- and internal elastic lamina, and total cross-sectional intimal area between the endothelial cell monolayer and the internal elastic lamina were measured. The intensities of staining for macrophages, SMCs, and collagen content within intimal tissue and medial layers were quantified as the average over 6 consecutive cross-sections and were expressed as a percentage of the total surface area per cuffed section.

### 2.5. Flow Cytometry

**Figure 1:**
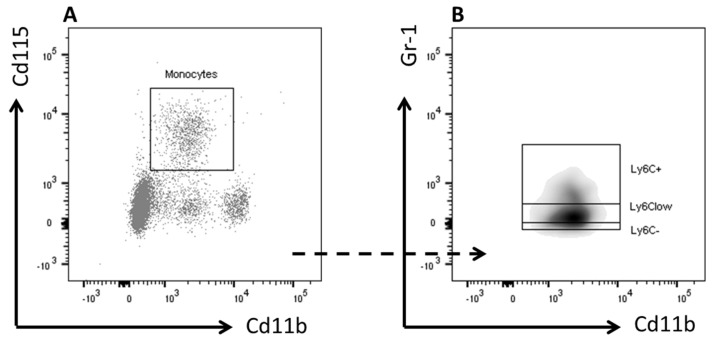
Gating strategy. Gating strategies for the analysis of (**A**) granulocytes, total monocytes; and (**B**) Ly6C^+^, Ly6C^low^, and Ly6C^-^ monocyte subsets in whole blood.

Circulating granulocytes and monocytes were analyzed using flow cytometry. After lysis of red blood cells, pelleted cells were re-suspended in FACS buffer and stained for 30 min at 4 °C in the dark with fluorescently labeled antibodies listed in Table 1. Cells were measured on an LSR II flow cytometer using Diva 6 software (BD Biosciences, San Jose, CA, USA). Data were analyzed using FlowJo software (Treestar, Ashland, OR, USA). Representative gating schemes are shown in Figure 1.

### 2.6. Plasma Total Cholesterol

Blood samples were collected in week 0, 3, and 5 after 4 h fasting (from 8:00 to 12:00 AM) via tail vein bleeding into chilled capillaries, and isolated plasma was assayed for total cholesterol (TC) using a commercially available kit (Roche Diagnostics, Mannheim, Germany). Cholesterol exposure was calculated as the cumulative exposure over the number of weeks fed either the control or the inulin-supplemented diet.

### 2.7. Statistical Analysis

Data are presented as means ± SEM. Normal distribution of the data was tested using D’Agostino-Pearson omnibus normality test, and data were compared in case of normal distribution with the unpaired Student’s *t*-test or in the case of not normally distributed data with the nonparametric Mann–Whitney U test. Differences in body weight and food intake were evaluated for statistical significance by two-way ANOVA followed by Sidak’s post hoc multiple comparison test. Correlation analysis was performed using linear regression analysis. The regression lines of the inulin supplemented mice versus control mice were compared to identify whether the correlations differed between the groups. First it was tested whether slopes of the lines differed and then whether intercepts of the lines differed. When the slopes and intercepts were not significantly different, linear regression analyses was performed on pooled data of both groups. *p* < 0.05 was considered as statistically significant. Analyses were performed using Graph Pad Prism version 7.0 (GraphPad Software, San Diego, CA, USA).

## 3. Results

### 3.1. Inulin Increased Atherosclerotic Lesion Formation and Outward Vascular Remodeling

**Figure 2:**
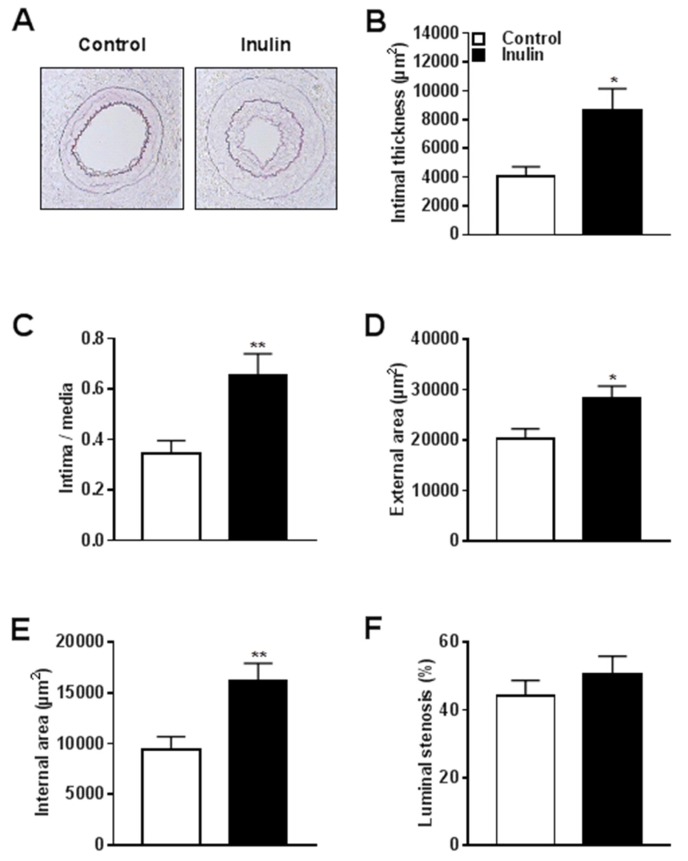
Inulin increased atherosclerotic lesion formation and outward vascular remodeling. (**A**) Representative cross-sections of the cuffed femoral arteries of *E3L* mice stained with Weigert’s elastin staining visualizing the elastic laminae; (**B**) Quantification of intimal lesion thickening; (**C**) Intima/media ratio; (**D**) External surface area; (**E**) Internal surface area; and (**F**) Percentage of luminal stenosis. Values are presented as means ± SEM (*n* = 11–13 mice per group). * *p* < 0.05, ** *p* < 0.01 vs. control.

We examined the effect of inulin on vascular pathology 14 days after polyethylene cuff placement around the femoral artery. Figure 2A shows a representative picture of lesion formation in control and inulin supplemented mice. Quantification of the intimal lesion demonstrated that inulin increased lesion surface area (µm^2^) by 72% compared to the control group (*p* = 0.01; Figure 2B). Since the total surface area (µm^2^) of the media was similar for both groups (Table 2), inulin significantly increased the intima/media ratio by 91% compared to controls (*p* = 0.01; Figure 2C). Outward vascular remodeling took place after inulin supplementation, as shown by increased external (+40% vs. control; *p* = 0.01; Figure 2D) and internal (+73% vs. control; *p* = 0.01; Figure 2E) surface areas. The percentage of luminal stenosis was not affected by inulin (Figure 2F). Compared to the control group, the surface area of the lumen was not changed by inulin (Table 2), which further indicates that inulin did not lead to inward vascular remodeling but rather induced outward vascular remodeling.

### 3.2. Inulin Induced Changes in Lesion Composition

To investigate whether inulin affected the lesion composition, we examined the medial and intimal lesion phenotype using (immuno)histochemical analysis. Consecutive cross-sections of the cuffed femoral arteries were stained with Sirius red for collagen, α-actin for SMCs, and MAC3 for the presence of macrophages in the surface area of the plaques. 

**Figure 3:**
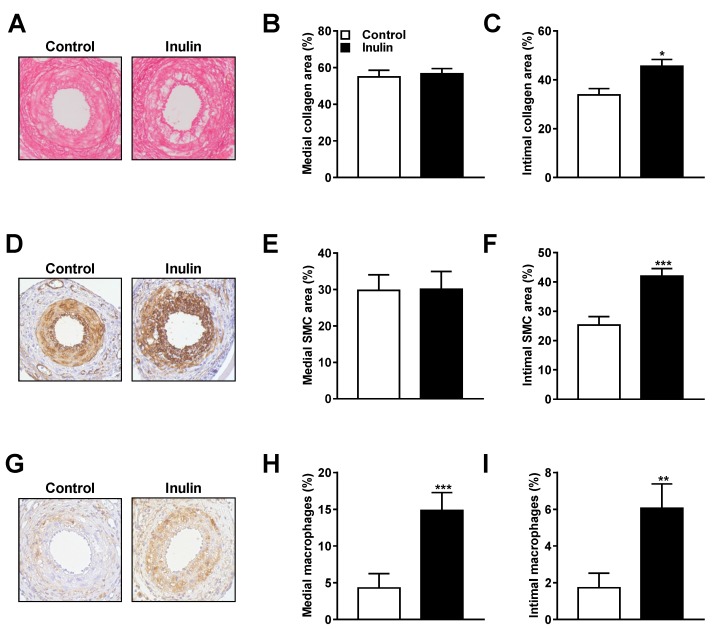
Inulin-induced changes in lesion composition. Representative cross-sections and quantitative analysis for medial and intimal lesion areas of the cuffed femoral arteries of *E3L* mice stained with (**A**–**C**) Sirius red for collagen; (**D**–**F**) α-actin for SMCs; and (**G**–**I**) MAC3 for macrophages. Values are presented as means ± SEM (*n* = 11–13 mice per group). * *p* < 0.05, ** *p* < 0.01, *** *p* < 0.001 vs. control.

Representative cross-sections of the cuffed femoral arteries stained for collagen are shown for both the control and the inulin group (Figure 3A). Inulin did not affect medial collagen area (Figure 3B), but increased the intimal collagen area (+35% vs. control; *p* = 0.01; Figure 3C). Figure 3D shows representative cross-sections of the cuffed femoral arteries stained for SMCs in both intervention groups. Likewise, inulin did not affect SMCs in the medial area (Figure 3E), but increased the area of intimal SMCs (+66% vs. control; *p* = 0.001; Figure 3F). Representative cross-sections stained for macrophages are shown in Figure 3G. Inulin substantially increased the intensity stained for macrophages of both the medial area (+247% vs. control; *p* = 0.001; Figure 3H) as well as the intimal lesion area (+259% vs. control; *p* = 0.002; Figure 3I). These data showed that inulin adversely affected lesion composition in hypercholesterolemic mice after perivascular cuff placement. 

### 3.3. Inulin did not Affect Blood Monocyte Composition but Increased Total Cholesterol Exposure

**Figure 4:**
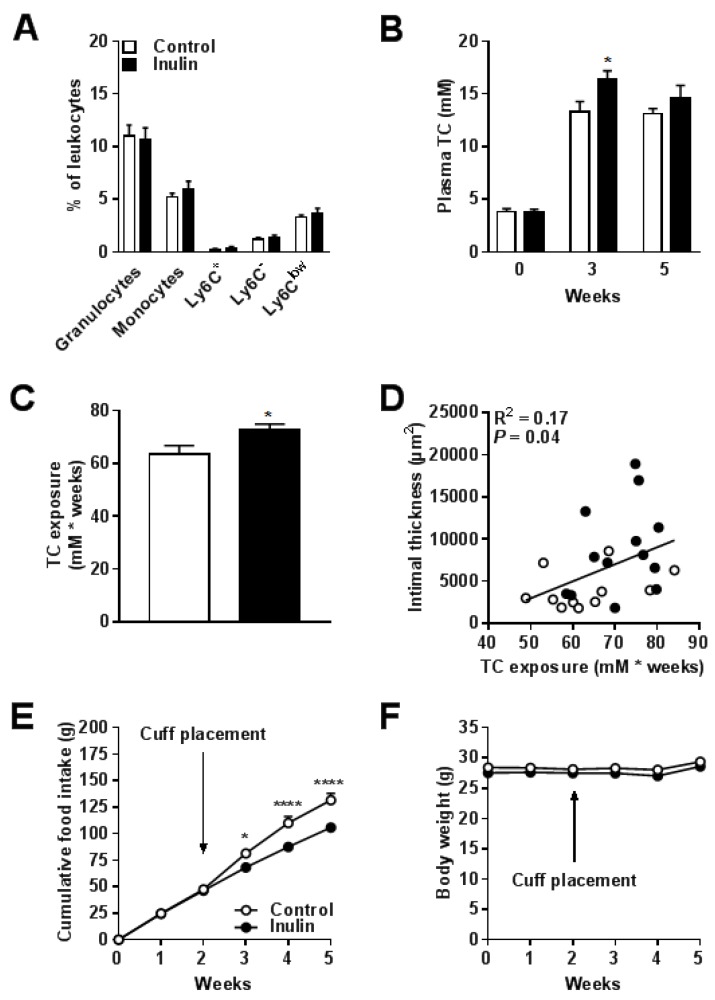
Inulin did not affect blood monocyte composition but increased total cholesterol exposure. (**A**) Circulating granulocytes, monocytes, and monocyte subsets Ly6C^+^, Ly6C^low^, and Ly6C^-^ are shown as a percentage of circulating leukocytes; (**B**) Plasma TC was analyzed in week 0, 3, and 5; and (**C**) Cumulative TC exposure was calculated over the entire intervention period of 5 weeks; (**D**) TC exposure was plotted against intimal thickness; (**E**) Cumulative food intake and (**F**) Body weight over the 5-week intervention period. The arrow indicates the time point at which the cuff was placed around the femoral artery. Values are presented as means ± SEM (*n* = 11–13 mice per group). * *p* < 0.05 vs. control.

The effect of inulin on blood monocyte composition was determined by flow cytometry. Inulin did not alter the percentages of circulating granulocytes, monocytes, and the monocyte subsets Ly6C^+^, Ly6C^low^, and Ly6C^-^ (Figure 4A). However, inulin increased plasma TC levels in week 3 (+23% vs. control; *P* = 0.02; Figure 4B), which overall led to an increased cholesterol exposure over the entire intervention period of 5 weeks (+14% vs. control; *p* = 0.03; Figure 4C). We performed regression analysis on TC exposure versus intimal thickness. Comparison of the regression lines indicated that slopes (F_slopes_ = 0.49; *p* = NS) and intercepts (*F*_intercepts_ = 3.98; *p* = NS) were similar for the control group and the inulin group (pooled data *R*^2^ = 0.17; *p* = 0.04; Figure 4D). This suggests that the aggravated lesion formation after inulin supplementation was driven by plasma TC. Finally, inulin decreased food intake (up to −17% in week 5 vs. control; *p* < 0.0001; Figure 4E) without affecting body weight (Figure 4F). Together, these data indicate that the mechanism behind the aggravated lesion formation seemed to be driven by cholesterol exposure.

## 4. Discussion

There are clear indications that dietary fibers, and specifically the prebiotic inulin, reduce cardiovascular risk factors such as systemic inflammation and hyperlipidemia [15,16,22,23,24]. However, in contrast to our expectations, we found that inulin aggravated atherosclerosis development in *E3L* mice. Inulin enlarged the intimal lesion thickness area as well as the collagen content and the percentages of macrophages and SMCs within the lesion. Furthermore, inulin increased outward vascular remodeling in these mice. The aggravated atherosclerosis development was likely explained by increased cholesterol exposure but not by alterations in blood monocyte composition.

In contrast to our results, Rault-Nania et al. [17] found that inulin-type fructans reduced atherosclerotic plaque formation by 35% in hypercholesterolemic male mice. However, this study was performed in APOE-deficient mice. Complete deficiency of APOE is associated with a systemic pro-inflammatory state [29]. In addition, APOE-deficient mice are characterized by severely disrupted LDL-receptor mediated lipoprotein remnant clearance and severe hypercholesterolemia [30]. In contrast, *E3L* mice express a dominant variant of human APOE characterized by a moderately disturbed LDL receptor mediated clearance [25]. These mice are highly responsive to diet-induced hyperlipidemia and atherosclerosis development and have been extensively used as preclinical model (review, see [31]). The differential effect of inulin on atherosclerosis development in APOE-deficient mice versus *E3L* mice is therefore likely mouse model-specific. Since *E3L* mice respond similarly as patients to a variety of anti-atherosclerotic interventions [31], we interpret our data to indicate caution with the application of inulin in humans.

We are not the first ones to show adverse effects of inulin on disease outcome. Miles et al. [32] reported that diets enriched with inulin did not protect, but further exacerbated the severity of dextran sulfate sodium (DSS)-induced colitis in mice. Two large randomized-controlled trials were performed in which patients with Crohn’s disease received either 15 g [33] or 20 g [34] oligofructose/inulin per day. They revealed increased severity of disease in the first study and withdrawal of 30% of patients in the second study due to adverse side effects. These studies suggested that the adverse effects of inulin were likely to be mediated via diverse interactions of inulin with the gut microbiota. However, the adverse effects of inulin were mainly found in combination with severe intestinal/colonic inflammation. This indicates that the effect of inulin on disease outcome might be context dependent. Moreover, it has previously been shown that exposure to diets high in cholesterol are able to serve as a precursor for intestinal inflammation in epithelial cells [35]. It therefore remains possible that a high-cholesterol diet facilitates intestinal inflammation and is associated with the detrimental effects of inulin on atherosclerosis in mice. The consideration that the context of diet affects disease outcome is supported by Goto et al. [36], demonstrating that inulin can either positively or negatively affect diarrhea and weight loss in mice, depending on the type of chow diet the mice were fed. It remains to be investigated whether the adverse effects of inulin are a consequence of different context dependent factors, e.g., diet and microbiota composition.

The mode of action of inulin has been shown to depend on inulin chain length. Vogt et al. [37] reported that short-chain inulin compared to long-chain inulin induced a more anti-inflammatory phenotype in PBMCs in vitro as determined by IL10/IL-12 cytokine production. In our study, we used long-chain inulin, but observed no effects on blood monocyte composition. The effects of short-chain versus long-chain inulin on atherosclerosis development in vivo remain to be investigated. 

In addition to inulin chain-length, the concentration of inulin added to the diet might influence disease outcome. We fed the mice a high-cholesterol diet supplemented with 10% inulin, which is a relatively high concentration of inulin. However, in another study by Parnell and Reimer [38], obese hyperlipidemic rats were given 10% inulin for a total period of 10 weeks, in which they established a decrease of 24% in circulating cholesterol levels. Although we cannot exclude different effects of inulin within various species, it remains to be determined whether other percentages of dietary inulin will result in lower plasma cholesterol levels in *E3L* mice. 

We observed increased outward vascular remodeling of the femoral artery in the inulin group. Inward vascular remodeling in arteries is an important determinant for lumen loss, whereas outward vascular remodeling can compensate for plaque accumulation in the arterial lumen [39]. Outward remodeling together with a preserved luminal area as observed in our study often indicates a more vulnerable plaque phenotype [40]. The plaque phenotype is determined by collagen turnover [41] and inflammation [42]. Indeed, inulin in our study resulted in changes in the composition of both the media and intima of the plaques, which indicates that the increased lesion formation after inulin supplementation was accompanied by more vulnerable plaques. The adverse effects of inulin on atherosclerosis development could not be explained by changes in blood monocyte composition. However, we cannot exclude the possibility that inulin might have modulated other systemic immune markers or that it has affected the immune status more subtly.

We found that inulin significantly increased plasma cholesterol levels and as a consequence exacerbated atherosclerosis development. The effect of inulin on plasma cholesterol levels might be mediated via interactions with the gut microbiota, by stimulating growth and/or activity of selective bacteria in the gut. We speculate that inulin can increase plasma cholesterol levels via modifications in the production of short-chain fatty acids (SCFAs) by the gut microbiota as suggested by previous studies [43,44]. In the colon, the SCFAs acetate and propionate are produced, absorbed, and transported via the portal vein to the liver [45]. In the liver, acetate is used as a substrate for *de novo* cholesterol and TG synthesis, while propionate inhibits cholesterol synthesis [46]. Variation in the propionate:acetate ratio that reaches the liver might therefore affect either hepatic stimulation or inhibition of *de novo* cholesterol and TG synthesis, resulting in differences in plasma cholesterol levels [47]. The notion that propionate:acetate ratios determine lipid metabolism is supported by a study by Weitkanut et al. [48], who found that an increased propionate:acetate ratio was associated with decreased hepatic expression of genes involved in lipogenesis and fatty acid elongation/desaturation of inulin-fed animals. Whether inulin in our study led to a decreased propionate:acetate ratio and therefore adversely affected plasma cholesterol levels and atherosclerosis development, remains to be investigated.

In conclusion, we found that inulin aggravated atherosclerosis development after placement of a cuff around the femoral artery in hypercholesterolemic *E3L* mice. This effect was accompanied by adverse changes in composition of both medial and intimal lesion areas, as well as increased outward vascular remodeling. The adverse effects of inulin on atherosclerosis development were mainly a result of increased plasma total cholesterol levels. Previous studies together with our data therefore raise the concern that inulin not always exert beneficial effects. It will be of importance for future research to decipher potential pathways and mechanisms induced by inulin under various conditions.

## Figures and Tables

**Table 1:** Antibodies used for flow cytometry.

Antibody	Fluorochrome	Dilution	Clone, Supplier
CD45.2	FITC	1:100	104, BioLegend
CD11b	Pacific Blue	1:150	M1/70, BioLegend
CD115-Biotin Streptavidin	n.a. PeCy5	1:100 1:100	AFS98, eBioScience SAV, eBioScience
Gr-1	PeCy7	1:1500	RB6-8C5

**Table 2:** Experimental measurements including vascular pathology, plasma monocytes, plasma cholesterol, body weight, and food intake.

**Vascular Pathology**	**Control (*n* = 11)**	**Inulin (*n* = 13)**	***p*-Value**
	**(Mean ± SEM)**	**(Mean ± SEM)**	
Intimal thickness (µm^2^)	4043 ± 689.5	8685 ± 1462	0.013 *
Intima/media	0.35 ± 0.05	0.65 ± 0.09	0.008 *
External area (µm^2^)	20303 ± 1942	28515 ± 2225	0.012 *
Internal area (µm^2^)	9383 ± 1288	16203 ± 1715	0.005 *
Luminal stenosis (%)	44.21 ± 4.56	50.72 ± 5.21	0.367
Lumen area (µm^2^)	5340 ± 961.1	7518 ± 1376	0.224
Medial area (µm^2^)	10920 ± 723.1	12312 ± 755	0.201
Medial collagen area (%)	54.88 ± 3.67	56.69 ± 2.76	0.692
Intimal collagen area (%)	33.79 ± 2.62	45.6 ± 2.74	0.011 *
Medial SMC area (%)	29.74 ± 4.29	30.1 ± 4.88	0.958
Intimal SMC area (%)	25.31 ± 2.81	41.93 ± 2.57	0.001 *
Medial macrophages (%)	4.299 ± 1.92	14.85 ± 2.43	0.001 *
Intimal macrophages (%)	1.73 ± 0.78	6.06 ± 1.33	0.002 *
**Plasma monocytes**	**Control (*n* = 11)**	**Inulin (*n* = 11)**	***p*-value**
	**(mean ± SEM)**	**(mean ± SEM)**	
Granulocytes (%)	10.55 ± 1.19	10.64 ± 1.14	0.956
Monocytes (%)	4.91 ± 0.39	6 ± 0.71	0.319
Ly6C^+^ (%)	0.18 ± 0.12	0.36 ± 0.15	0.635
LyC6^−^ (%)	1 ± 0.14	1.36 ± 0.24	0.23
Ly6C^low^ (%)	3.18 ± 0.26	3.63 ± 0.51	0.737
**Plasma cholesterol**	**Control (*n* = 11)**	**Inulin (*n* = 13)**	***p*-value**
	**(mean ± SEM)**	**(mean ± SEM)**	
Plasma TC t = 0 (mM)	3.83 ± 0.29	3.79 ± 0.26	0.924
Plasma TC t = 3 (mM)	13.28 ± 1	16.33 ± 0.85	0.024 *
Plasma TC t = 5 (mM)	13.12 ± 0.5	14.65 ± 1.14	0.738
TC exposure (mM*Weeks)	63.54 ± 3.20	72.55 ± 2.38	0.03 *
**Body weight and Food intake**	**Control (*n* = 11)**	**Inulin (*n* = 13)**	***p*-value**
	**(mean ± SEM)**	**(mean ± SEM)**	
Body weight t = 0 (g)	28.42 ± 0.46	27.53 ± 0.54	0.832
Body weight t = 1 (g)	28.35 ± 0.49	27.61 ± 0.56	0.921
Body weight t = 2 (g)	28.12 ± 0.55	27.49 ± 0.55	0.962
Body weight t = 3 (g)	28.28 ± 0.54	27.47 ± 0.56	0.884
Body weight t = 4 (g)	28.01 ± 0.49	27.02 ± 0.55	0.754
Body weight t = 5 (g)	29.35 ± 0.54	28.59 ± 0.61	0.907
Cumulative food intake t = 1 (g)	24.81 ± 0.78	24.4 ± 1.7	>0.999
Cumulative food intake t = 2 (g)	47.56 ± 1.51	46.3 ± 2.4	>0.999
Cumulative food intake t = 3 (g)	81.33 ± 3.22	68.16 ± 2.71	0.035 *
Cumulative food intake t = 4 (g)	109.94 ± 6.11	87.39 ± 2.76	<0.0001 *
Cumulative food intake t = 5 (g)	131.43 ± 6.52	105.81 ± 2.78	<0.0001 *

* *p* < 0.05 Control vs. Inulin. SMC = smooth muscle cell; TC = Total cholesterol exposure.
